# Immediate Release Formulation of Inhaled Beclomethasone Dipropionate-Hydroxypropyl-Beta-Cyclodextrin Composite Particles Produced Using Supercritical Assisted Atomization

**DOI:** 10.3390/polym14102114

**Published:** 2022-05-23

**Authors:** Hsien-Tsung Wu, Yao-Hsiang Chuang, Han-Cyuan Lin, Tzu-Chieh Hu, Yi-Jia Tu, Liang-Jung Chien

**Affiliations:** Department of Chemical Engineering, Ming Chi University of Technology, 84 Gungjuan Rd., Taishan Dist., New Taipei City 24301, Taiwan; M07138123@mail2.mcut.edu.tw (Y.-H.C.); M08138104@mail2.mcut.edu.tw (H.-C.L.); M09138112@mail2.mcut.edu.tw (T.-C.H.); M09138206@mail2.mcut.edu.tw (Y.-J.T.); ljchien@mail.mcut.edu.tw (L.-J.C.)

**Keywords:** supercritical assisted atomization, hydroxypropyl-beta-cyclodextrin, beclomethasone dipropionate, in vitro dissolution, in vitro atomization

## Abstract

In this study, the enhanced solubilization performance of a poorly soluble drug, beclomethasone dipropionate (BDP), was investigated using hydroxypropyl-β-cyclodextrin (HP-β-CD) and ethanol. The enhanced solubility of the drug was determined using the phase solubility method and correlated as a function of both HP-β-CD and ethanol concentrations. The effective progress of drug solubility originated from the formation of cyclodextrin and BDP inclusion complexes and increase in the lipophilicity of the medium, by aqueous ethanol, for hydrophobic BDP. BDP and HP-β-CD composite particles were produced using supercritical assisted atomization (SAA) with carbon dioxide as the spraying medium, 54.2% (*w/w*) aqueous ethanol as the solvent, and an optimal amount of the dispersion enhancer leucine. The effect of the mass ratio of HP-β-CD to BDP (*Z*) on the in vitro aerosolization and in vitro dissolution performance of BDP–HP-β-CD composite particles was evaluated. The aerosolization performance showed that the fine particles fraction (*FPF*) of the composite particles increased with increasing mass ratio. The water-soluble excipient (HP-β-CD) effectively enhance the dissolution rate of BDP from composite particles. This study suggests that BDP–HP-β-CD composite particles produced using SAA can be employed in immediate-release drug formulations for pulmonary delivery.

## 1. Introduction

Beclomethasone dipropionate (BDP) is a poorly water-soluble glucocorticosteroid drug (<1 μg/mL) used as a nasal spray or dry powder inhalation for the treatment of respiratory and asthmatic diseases. Pulmonary delivery of active pharmaceutical ingredients (APIs) has been extensively investigated because of its superior compatibility with other routes of administration and is mainly provided by three types of inhaler devices: nebulizer, pressurized metered dose inhalers (pMDIs), and dry powder inhalers (DPIs). DPIs have many advantages, including no requirement of a propellant, portability, convenience of use, high dose delivery, and high chemical stability of the solid state. Water-soluble hydroxypropyl-β-cyclodextrin (HP-β-CD) has been specified by the FDA as an inactive pharmaceutical ingredient and is commonly used in oral, rectal, dermal, ocular, and parenteral formulations [1]. HP-β-CD and poorly water-soluble drugs can form water-soluble inclusion complexes and promote drug molecule permeability and bioavailability [2,3,4]. In term of cytotoxicity, HP-β-CD has been suggested to be safe for application in human airway epithelial Calu-3 and A549 cells in vitro, demonstrating its potential for use in commercial dry powders as inhalation formulations [2,5]. In environments protection issues, Gao et al. [6,7] elaborated on the inclusion complex of H-β-CD and insoluble drugs, such as difenoconazole and thiophanate-methyl, to enhance the solubility of drug and reduce the amount of their residues in the environment during pesticide formulation.

Conventional dry powder inhaler formulations often blend fine cohesive drug particles and coarse carrier particles, which are used to improve flowability and aerosolization properties. This formulation improves dose accuracy. However, the detachment of fine drug particles from the carrier particles is insufficient and results in poor drug delivery to the lung. Gilani et al. [8] investigated the in vitro pulmonary deposition of inhalable formulations of BDP blended with different carriers, including spray-dried lactose/poly(ethylene glycol) (lactose/PEG) particles with asperity surfaces as fine carriers and commercial lactose as a coarse carrier. The improved aerosolization performance of the lactose/PEG carrier resulted in easier detachment of the BDP microparticles from the smaller asperity surface of the fine carrier of lactose/PEG than from the large asperity surface of commercially coarse lactose during aerosolization. Cabral-Marques and Almeida [9] evaluated the aerodynamic performance of BDP/γ-cyclodextrin composite particles produced using a spray dryer and physically mixed with three carriers (lactose, trehalose, and respitose), and suggested that finer carriers of lactose and trehalose exhibited better aerodynamic performance.

Over the past few decades, a combination of API and different functional excipients to improve the physical properties and aerosolization performance of API drugs without physically blending with coarse or fine carrier has been reported. Vartiainen et al. [2] used HP-β-CD as a solubilizer and leucine (LEU) as a coated agent to enhance the dissolution and aerosol performance of corticosteroid formulations produced using an aerosol flow reactor. Spray drying is a commonly used method for inhalable dry powder formulations of API and functional excipients. The co-spray drying of raffinose, trehalose, and HP-β-CD composites resulted in improved physical stability and aerodynamic behavior compared to raffinose and trehalose spray drying alone [10]. Mohtar et al. [11] suggested that the spray-dried complex of sulfobutylether-β-cyclodextrin (SBE-β-CD) and fisetin with leucine improved the solubility of the drug and delivered a high amount of fisetin to the deep lung region. Suzuki et al. [12] developed a roflumilast dry powder inhaler formulation using spray drying with HP-β-CD as an excipient/particle matrix and ethanol as a solvent. 

Supercritical assisted atomization (SAA) has been proposed as an alternative to the abovementioned conventional process for micronization owing to its many advantages, including high yield [13], continuous scalability [14], and processing of thermosensitive proteins or low melting materials [15,16,17]. Recently, HP-β-CD has been used as a carrier or excipient in drug coprecipitates produced using SAA, including curcumin/HP-β-CD and propolis/HP-β-CD composites [18,19]. However, the formulation of BDP/HP-β-CD composite for pulmonary delivery has not yet been explored. The aim of the present study was to develop a novel immediate-release DPI formulation of BDP/HP-β-CD/LEU composite particles produced using SAA. The enhanced solubility of BDP resulting from HP-β-CD and ethanol/co-solvent was determined by the phase solubility method [20], which provides the basis for the selection of optimal SAA operation parameters. Thus, HP-β-CD was used as a solubilizer or excipient to enhance the dissolution rate of poorly water-soluble drugs and LEU was used as a dispersion enhancer to improve the aerosolization performance of inhaled dry powder. The in vitro aerosolization and dissolution performances of the BDP/H-β-CD/LEU composite particles were evaluated using an Andersen cascade impactor (ACI) and a dissolution tester, respectively. X-ray diffraction (XRD) and Fourier transform infrared spectroscopy (FTIR) were used to characterize the crystalline and structural properties of the BDP/H-β-CD composite particles. Differential scanning calorimeter (DSC) was used to verify the thermal behavior of amorphous inclusion complexes.

## 2. Materials and Methods

### 2.1. Materials

Hydroxypropyl-beta-cyclodextrin (HP-β-CD, 99.9% purity, degree of substitution: ~0.8, MW = 1460 g/mol) and _L_-leucine (99.9% purity) were purchased from Sigma Aldrich, St. Louis, MO, USA. Ethanol and methanol (99.9% purity, high-performance liquid chromatography grade) were purchased from Acros, Branchburg, NJ, USA. Beclomethasone dipropionate (BDP, 99% purity, C_28_H_37_ClO_7_, MW = 521 g/mol) was purchased from Tokyo Chemical Industry, Tokyo, Japan. Carbon dioxide (99.9% purity) and nitrogen (99.9% purity) were purchased from Yung-Ping Gas Co., Taipei, Taiwan. All chemicals were used without further purification. A Millipore Milli-Q water purification system was used to obtain deionized water with a resistivity of 18 MΩ·cm at 25 °C.

### 2.2. Phase Solubility Studies

A phase solubility study was conducted according to the method reported by Higuchi and Connors [20] to investigate the influence of cyclodextrin (CD) and ethanol as co-solvent on the solubilization of BDP. A series of aqueous ethanol (0–54.2%, *w*/*w*, or 0–10.3 M) and HP-β-CD (0–5%, *w*/*v*, or 0–34 mM) were obtained by adding ethanol and HP-β-CD to deionized water. The solubilities of BDP in aqueous systems containing a co-solvent, HP-β-CD, or combined co-solvent–HP-β-CD were measured using the phase solubility method. The phase solubility samples were prepared by adding excess amount of BDP to 10 mL mixed solvent in a test tube and were filtered with a 0.25 μm membrane under constant shaking at 25 °C for 72 h. The filtrates were diluted after an optimum time and then analyzed by HPLC to quantify BDP using UV detection at 254 nm [21]. Phase solubility profiles were obtained as the concentration of solubilized BDP (mM) plotted against different concentrations of cyclodextrin (mM) in the same solvent. The slope and y-intercept (*S*_0_) of the straight lines of the phase solubility profiles are used to calculate the stability constant (*K_S_*) and complexation efficiency (*CE*) as Equations (1) and (2):

(1) $$K_{S}=\frac{slope}{S_{0}\left(1-slope\right)}$$

where *S*_0_ is the intrinsic drug solubility, that is, the solubility of BDP in pure water when no cyclodextrin is present.

(2) $$CE=\frac{slope}{1-slope}$$

### 2.3. HPLC Analysis of BDP

High-performance liquid chromatography (HPLC; Varian, model 210, Palo Alto, CA, USA) was used to quantify BDP. A Quasar C18 column (5 μm, 150 mm × 4.6 mm, PerkinElmer, Shelton, CT, USA) was used as the stationary phase, with 93% methanol and 7% water as the mobile phase at a flow rate of 1.0 mL/min. The sample injection volume was 20 μL with UV detection at 254 nm; HP-β-CD and leucine had no absorbance at this wavelength. The calibration curves were plotted with the peak area of the analyte against the corresponding concentration (0.05 to 50 μg/mL) using linear regression analysis. The linear relationship between the peak areas and the concentration of BDP was *y* = 65.142*x* + 10.459 (*R^2^* = 0.9999), where *x* is the concentration (μg/mL) and *y* is the peak area (mV·s).

### 2.4. Production of Drug–HP-β-CD Composite Particles

A schematic diagram of the SAA apparatus and the experimental procedure have been provided elsewhere [22]. The apparatus consisted of three main chambers (saturator, precipitator, and separator) and three feeding lines (drug–HP-β-CD solution, CO_2_, and N_2_). Two high-pressure liquid pumps were used to deliver CO_2_ and the drug–HP-β-CD solution. Using a mass flow controller, the N_2_ flow was controlled from a cylinder, heated in an electric heat exchanger, and then sent to the precipitator to assist in the evaporation of liquid droplets.

The experimental procedure is briefly described as follows. The saturator temperature (*T_S_*) and the volumetric flow rate of CO_2_ were preset. The N_2_ flow rate was 1.0 Nm^3^/h. Once a steady state was achieved, the drug–HP-β-CD solution was introduced into the saturator via a pre-heated water bath at a flow rate of 3 mL/min. The CO_2_ mixture dissolved in the drug–HP-β-CD solution and obtained by the saturator was sprayed through an injection nozzle (inside diameter of 130 μm) to atomize the liquid droplets in the precipitator. After contact of the droplet solution with heated N_2_ and the consequent evaporation of the solvent from the droplets, drug–HP-β-CD composites were formed due to supersaturation of the solute. The samples were collected from the precipitator and observed using a field emission scanning electron microscope (FESEM, model 6500, JEOL, Tokyo, Japan). 

The particle size distribution (*PSD*) of the drug–HP-β-CD composite particles was determined using a dynamic light scattering (DLS) particle analyzer (Zatasizer Nano ZS90, Malvern, UK). As described previously [23], the particles were suspended in petroleum oil at 293.15 K and sonicated for 1 min. The *PSD* was calculated by applying the Mie theory, using a refractive index of *n* = 1.520 for HP-β-CD [24]. The arithmetic and mass-weighted mean particle diameters, *d_no_* and *d*_4,3_, respectively, were calculated from the equations $d_{no}=\overset{i}{\underset{i=1}{\sum }}x_{i}D_{i}$ and $d_{4,3}=\frac{\sum_{i=1}^{i}x_{i}D_{i}^{4}}{\sum_{i=1}^{i}x_{i}D_{i}^{3}}$, where *x* represents the number fraction of the particles. All precipitation experiments were performed in triplicate (*n* = 3). The average yield of the resulting particles during SAA was 80%. The weight loss was attributed to the adherence of a fraction of the microparticles to the walls of the precipitator and insides the filter pores.

### 2.5. Solid-State Characterization

The X-ray diffraction (XRD) patterns of the produced powders were recorded using an X’Pert Pro X-ray powder diffractometer (PANalytical, Almelo, The Netherlands) between 2θ values of 5° and 50° at a scan rate of 0.02°/s. The infrared (IR) spectra of the samples were recorded using a Fourier transform infrared spectrophotometer (Thermo Scientific Nicolet iS5 FTIR Spectrometer, Waltham, MA, USA) with an attenuated total reflection (ATR) element. The IR spectra were recorded from 400 to 4000 cm^−1^. Thermograms of the composite samples were obtained using a differential scanning calorimeter (LT-DSC, Netzsch 204 Fl Phoenix, Selb, Germany). Samples weighing 3–10 mg were placed in aluminum pans, which were then sealed and heated from 298.2 to 553.2 K at a rate of 10 K/min in a N_2_ atmosphere. 

The bulk density (*ρ_buk_*) and tapped density (*ρ_tap_*) of the drug–HP-β-CD powder were measured as descriptors of the bulk powder cohesiveness and flow properties. Each drug–HP-β-CD powder sample was placed in a 5 mL cylinder, and after recording the initial volume (bulk volume), the cylinder was tapped 1250 times (automated tap density analyzer, Auto top 02106-60-1, Quantachrome, Boynton Beach, FL, USA) and the new volume was recorded (tapped volume). The number of taps was 1250, following the recommendation of the European Pharmacopoeia. The *ρ_buk_* and *ρ_tap_* values were calculated as the powder weight over the powder bulk volume (*n* = 0) and tapped volume (*n* = 1250), respectively. The flowability of drug–HP-β-CD powder was estimated using the Hausner ratio (*H_R_* = *ρ_tap_*/*ρ_buk_*).

### 2.6. In Vitro Aerosol Performance Determined Using an ACI

The aerosol behavior of the drug–HP-β-CD composite was determined using a HandiHaler (Boehringer Ingelheim, Ingelheim, Germany) coupled through an induction port (United States Pharmacopeia (USP) sampling inlet) to an Andersen cascade impactor (ACI, TE-20801, Tisch, Cleves, OH, USA) operated at a flow rate of 60 L/min. Hydroxypropyl methylcellulose capsules (size 3) were filled with the sample powder (20.0 ± 0.5 mg) and placed into the HandiHaler. The airflow rate for ACI was adjusted to 60 L/min using a critical flow controller (TPK 2000, Copley, UK). This critical sonic flow was maintained (*P_3_*/*P_2_* < 0.5, where *P_3_* and *P_2_* are the inlet and outlet pressures of the controller, respectively) and the flow rate was assumed to be stable. The aerodynamic cut-off diameter of each stage of the ACI was calibrated by the manufacturer as follows: stage 1, 8.6 μm; stage 2, 6.5 μm; stage 3, 4.4 μm; stage 4, 3.3 μm; stage 5, 2.0 μm; stage 6, 1.1 μm; stage 7, 0.54 μm; and stage 8, 0.25 μm. The drug–HP-β-CD composites deposited at each stage were assayed by HPLC (Varian, model 210, Palo Alto, CA, USA). The emitted dose (*ED*) was determined as the difference between the initial mass of the powder loaded into the capsules (i.e., the total dose, *TD*) and the remaining mass of the powder in the capsules following aerosolization. The *ED* fraction (%) was used to express the percentage of *ED* based on the *TD* used. The fine particle dose (*FPD*) was defined as the quantity of particles with aerodynamic diameters <5 μm, and the dose deposited in stages 3–8 of the ACI assay was obtained. The fine particle fraction (*FPF*, %) was expressed as the percentage of *FPD* to *TD*. In addition, to calculate the mass median aerodynamic diameter (*MMAD*), the cumulative percentage of the powder mass smaller than the stated aerodynamic diameter of the impactor stages from 1–8 was calculated and plotted against the effective cut-off diameter on a log probability plot. The *MMAD* of aerosol particles was calculated using the method described by O’Shaughnessy and Raabe [25]. The in vitro aerosolization was evaluated in triplicate (*n* = 3) under ambient conditions with a relative humidity of 40 ± 5%, and the results were used to calculate the standard deviation for each set of experimental conditions.

### 2.7. Drug Content Determination and In Vitro Dissolution Tests

Accurately weighed 5 mg samples of BDP–HP-β-CD composite microparticles were suspended in 20 mL of ethanol and ultrasonicated for 2 h to completely dissolve the BDP. The dissolved samples were centrifuged at 10,000 rpm for 10 min, and the supernatant was filtrated through a 0.25 μm membrane filter. The amount of BDP in the filtered solution was determined using HPLC. The wavelength for the assay was set at 254 nm; HP-β-CD and leucine showed no absorbance at this wavelength. The drug content (%, *w/w*) of the composite particles was determined by calculating the ratio between the weights of BDP in the filtered solution and BDP–HP-β-CD composite particles. All measurements were conducted in triplicate (*n* = 3). 

The dissolution test was performed according to the USP guidelines using apparatus 1 (basket method, DT6, Shin Kwang, Taiwan). The powders were filled into hard gelatin capsules of size 0, each containing 5 mg of BDP-equivalent powder. A dissolution study of the as-received BDP and BDP–HP-β-CD composite particles was performed in 1000 mL of 0.05 M PBS maintained at 310.2 ± 0.5 K. The rotational speed of the basket was set at 50 rpm. At suitable time intervals, 5 mL of the dissolution medium was removed and immediately replaced with a fresh medium. Dissolved BDP was analyzed using an HPLC spectrophotometer at 254 nm. All measurements were performed in triplicate (*n* = 3). The dissolution profiles of the as-received BDP and the mass ratio (*Z*) between HP-β-CD and BDP of the composite particles were used to understand the drug release properties.

## 3. Results and Discussion

### 3.1. Phase Solubility Study

**Figure 1:**
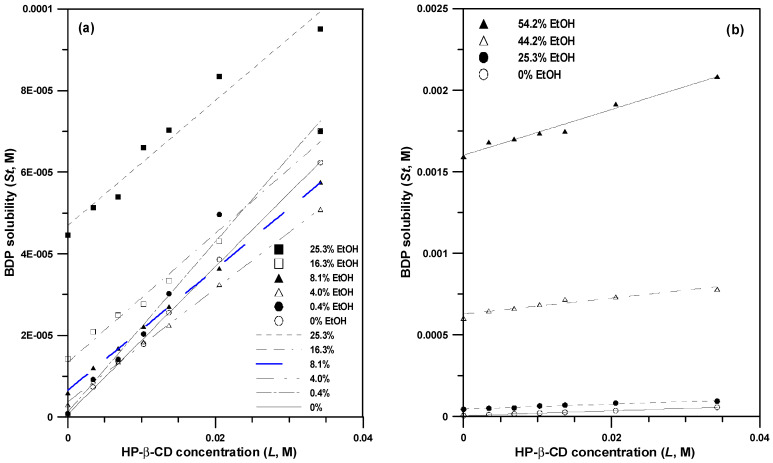
Phase solubility profiles of BDP in HP-β-CD solutions with increasing concentration of ethanol: (**a**) 0–25.3%, *w*/*w* EtOH; (**b**) 25.3–54.2%, *w*/*w* EtOH.

BDP is a practically insoluble drug; in pure water, the solubility of BDP is in the range 0.1–2.4 μg/mL (0.12 ± 0.04 μg/mL [26], 2.17 μg/mL [27], 2.38 μg/mL [28], 0.16 μg/mL [29]). The different solubilities of BDP might result from the different measurement methods, crystal forms, and particle sizes of the drug [30]. In this study, the solubility of BDP in pure water was determined as 0.28 ± 0.10 μg/mL (5.28 × 10^−^^4^ mM) after multiple measurements (*n* = 6). Water-soluble HP-β-CD and BDP can form drug/CD inclusion complexes that enhance the solubility of poorly soluble drugs. Phase solubility studies were conducted to evaluate the solubility efficiency of HP-β-CD and co-solvent (ethanol). Figure 1 shows the phase solubility diagrams of BDP in 0–54.2% (*w*/*w*) aqueous ethanol as the inclusion medium, that is, the concentration of solubilized BDP (mM) was determined in these different mixtures with increasing concentrations of cyclodextrin (mM). Owing to the addition of organic solvents, the hydrogen bonds between water molecules are broken, and the lipophilicity of the medium increases, facilitating the solubilization of hydrophobic compounds such as BDP. The solubility of BDP was further enhanced by the addition of HP-β-CD in aqueous ethanol.

The phase solubility profiles of BDP in all HP-β-CD solutions exhibit a Higuchi A_L_-type phase solubility diagram [20]. The performance of cyclodextrin in solubilizing a given drug is frequently evaluated by comparing its stability constant (*K_S_*) and complexation efficiency (*CE*). The *Ks* and *CE* values from the phase solubility profile of BDP in pure water as the inclusion medium are 3488 M^−1^ and 0.0018, respectively, indicating a good combination of BDP and H-β-CD in this study. In comparison, Malaekeh-Nikouei et al., previously reported these values to be 682 M^−1^ and 0.003, respectively [28]. While the previous *CE* value is comparable to that obtained in this study, the *Ks* value in this study is five times that reported previously; this difference can be attributed to the difference in intrinsic solubility (*S*_0_) between the studies. *CE* is the ratio between the cyclodextrin complex and free cyclodextrin, and it is more reliable than the stability constant (*K_S_*), because it is independent of the intrinsic solubility and *y*-intercept of the phase solubility profiles [31]. Table 1 shows the *CE* values determined from the phase solubility diagram of BDP in HP-β-CD solutions with varying concentrations of the co-solvent. At low co-solvent concentrations, *CE* decreases with increasing concentration of ethanol, indicating that the increment in solubility is reduced. This can be explained as follows. As the environment becomes more hydrophobic, the guest (BDP) tends to dissolve in the surrounding medium or dissolve in the medium as a CD/BDP complex. This phenomenon has been reported in many studies, regardless of the type of CD, and destabilization of ethanol as a co-solvent has been observed [32,33].

**Figure 2:**
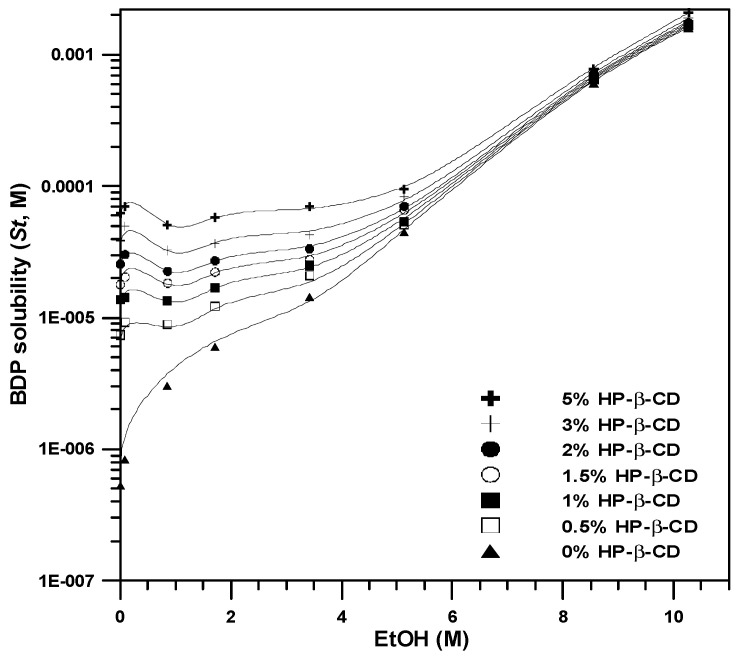
Dependence of BDP solubility on ethanol in ethanol–HP-β-CD solutions.

Figure 2 shows the phase solubility profiles of aqueous ethanol solutions with different concentrations (0, 0.4, 4.0, 8.1, 16.3, 25.3, 44.2, and 54.2% *w*/*w* EtOH) as the inclusion medium. The lowest solubility of BDP in the inclusion medium is approximately 4.0–8.1% EtOH, and BDP solubility increases with increasing ethanol concentration. These results are consistent with the minimum *CE* value in the inclusion medium of 4.0% EtOH (Table 1), which can be explained as follows. The increase in solubility at low ethanol concentrations is caused by the formation of a drug–CD–co-solvent ternary inclusion compound. The subsequent decrease in the solubility of the drug is caused by the dissociation of the drug–CD binary inclusion compound, that is, the aforementioned co-solvent destabilization effect. Subsequently, the solubility of the drug increases and the log-linear model represents the solubilization effect of the co-solvent. 

### 3.2. Total Drug Solubility of BDP

He et al. [34] established a mathematical model that can describe and explain the combination effect of a co-solvent and HP-β-CD on the solubilization of a drug. The total concentration of the drug in the aqueous solution consists of the free drug, drug–CD binary complex, and drug–co-solvent–CD ternary complex. The total drug solubility as a function of both cyclodextrin and co-solvent concentrations with five constants (*σ*, *K_b_*, *K_t_*, *ρ_b_*, and *ρ_t_*) is described by Equation (3).

(3) $$\frac{\left[D_{t}\right]}{\left[S_{0}\right]}=10^{\sigma \left[C\right]}+K_{b}\left[L\right]10^{\left(\sigma -\rho_{b}\right)\left[C\right]}+K_{t}\left[L\right]\left[C\right]10^{\left(\sigma -\rho_{t}\right)\left[C\right]}$$

where [*D_t_*] is the total apparent drug solubility; [*C*] and [*L*] are the concentrations of the co-solvent and cyclodextrin, respectively; *σ* is the co-solvent solubilization power; *K_b_* and *K_t_* are the formation constants for the binary and ternary complexes, respectively; and *ρ_b_* and *ρ_t_* are the co-solvent destabilizing powers for the binary and ternary complexes, respectively.

To determine the five constants (*σ*, *K_b_*, *K_t_*, *ρ_b_*, and *ρ_t_*), the data are treated using the method described by Li et al. [35]. When the concentration of CD ([*L*]) is 0, Equation (3) can be rearranged as

(4) $$\log\left[D_{t}\right]=\log\left[S_{0}\right]+\sigma \left[C\right];$$

**Figure 3:**
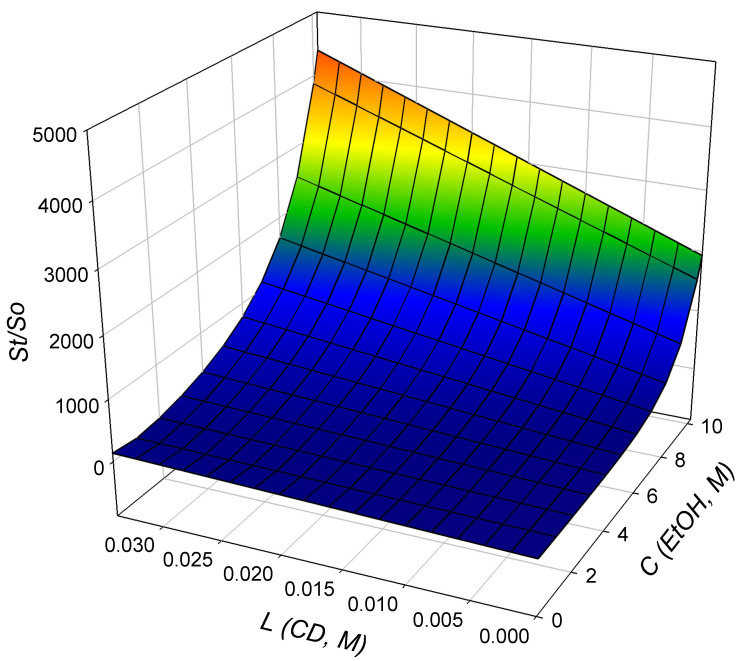
Correlated diagram of BDP solubility in HP-β-CD solutions with Equation (3) and parameters enlisted in Table 2.

*σ* can be determined from the slope of the phase solubility profile. When the concentration of ethanol ([*C*]) is 0, Equation (3) can be rearranged as

(5) $$\left[D_{t}\right]=K_{b}\left[S_{0}\right]\left[L\right]+\left[S_{0}\right],$$

and *K_b_* can be calculated from the slope and intercept of the phase solubility profile. When the concentration of ethanol is very low (e.g., [*C*] = 0.4%), the presence of ethanol does not influence the complexation process, and *ρ_b_* and *ρ_t_* can be assumed to be 0. Therefore, Equation (3) can be rearranged as

(6) $$\frac{\left[D_{t}\right]}{\left[S_{0}\right]}=10^{\sigma \left[C\right]}+K_{b}\left[L\right]10^{\sigma \left[C\right]}+K_{t}\left[L\right]\left[C\right]10^{\sigma \left[C\right]},$$

and *K_t_* can be calculated according to this Equation (6) with the known values of σ and *K_b_*. Then, *ρ_b_* and *ρ_t_* are calculated using a nonlinear regression process using data from the phase solubility profiles. All experimental data from the phase solubility profiles in this study ([*C*] = 0–54.2% (*w*/*w*), [*L*] = 0–5% (*w*/*v*)) were correlated using Equation (3), and the correlated parameters are shown in Table 2. The total solubility of BDP in solution is shown in Figure 3 as a function of ethanol concentration as well as HP-β-CD concentration, which is well correlated with the experimental data of the phase solubility experiments (*R^2^* = 0.93). A 54.2% (*w*/*w*) aqueous ethanol (10.27 M) solution containing 5% (*w*/*v*) HP-β-CD (34 mM) increases the solubility of BDP in solution to 1087 μg/mL (2.10 mM), which is 4000 times of the intrinsic BDP solubility. Therefore, 54.2% (*w*/*w*) aqueous ethanol has been used in subsequent SAA experiments to prepare HP-β-CD and drug composite particles.

### 3.3. In Vitro Aerosolization Performance of the Drug–HP-β-CD Formulation

**Figure 4:**
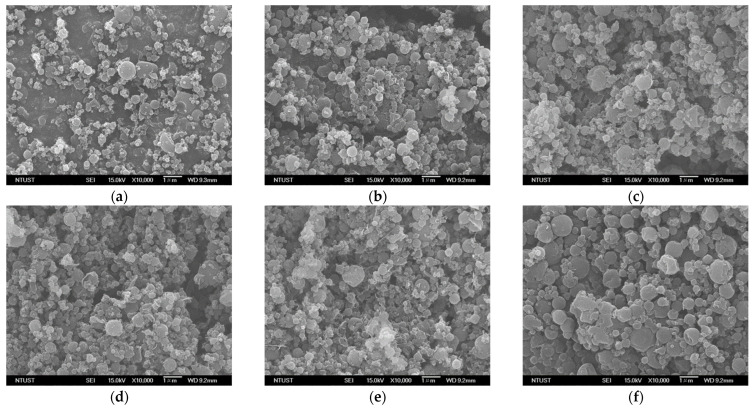
FESEM images of drug–excipient composites produced through SAA at different HP-β-CD/BDP mass ratio (*Z*): (**a**) *Z* = 15 (leucine free), (**b**) *Z* = 15, (**c**) *Z* = 20, (**d**) *Z* = 25, (**e**) *Z* = 30, (**f**) *Z* = 35.

The solvent concentration and optimal conditions for the production of drug–HP-β-CD composites via SAA were adopted from the phase solubility study and a previous study on the formation of fine spherical HP-β-CD particles with excellent aerosolization performance [36]. These conditions include 54.2% (*w*/*w*) aqueous ethanol as the solvent, mass flow ratio (*R*) of 1.95 between CO_2_ and the ethanol solution, precipitator temperature and saturator temperature of 373.15 K and 353.15 K, respectively, and addition of the dispersion enhancer (LEU) with an optimal concentration of 13.0 mass% (100 × LEU/(LEU+HP-β-CD)). Prior to the preparation of the drug–HP-β-CD composite particles, the function of the dispersion enhancer LEU was confirmed. The HP-β-CD concentration (*C_HP_*) of the drug–excipient solution was fixed at 10 mg/mL, and the HP-β-CD-to-drug mass ratio (*Z* = HP-β-CD/BDP) was chosen as 15. FESEM images of the composite particles without LEU and after addition of 13 mass% leucine at the same mass ratio are shown in Figure 4a,b, respectively. The particle sizes and shapes are not significantly different, but fine wrinkled surface features of the composite particles evident with the addition of 13 mass% leucine enhance their aerosolization performance. The in vitro aerosolization performances of two ternary drug–excipient formulation composites (BDP–HP-β-CD-LEU) with 1 mass% (*C_LEU_* = 0.1 mg/mL) and 13.0 mass% LEU (*C_LEU_* = 1.5 mg/mL) produced via SAA are presented in runs #B2 and #B3, respectively, in Table 3. As expected, the sample powder with 13.0 mass% LEU (run #B3) possesses a higher fine particle fraction (*FPF* = 41.8 ± 2.3%) than the sample with 1 mass% LEU (*FPF* = 25.5 ± 1.0%) and a lower mass median aerodynamic diameter (*MMAD*) and Hausner ratio (*H_R_*) than the sample with 1 mass% LEU. This verifies the function of the dispersion enhancer LEU in ternary drug–excipient formulation composites (BDP–HP-β-CD-LEU). Therefore, drug–excipient composite particles with the addition of 13 mass% LEU were produced through SAA, and the composition of the ternary drug–excipient solution is listed in Table 3.

The effect of the mass ratio of HP-β-CD to drug (*Z* = HP-β-CD/BDP) on the aerodynamic performance of the CD/LEU/BDP three-component composite particles is presented in Table 3 (runs #B3–#B7). Figure 4b–f show the FESEM images of composite particles with different mass ratios (*Z*) produced by SAA process. The mean particle size of the composite particles increased slightly with the mass ratio. The reason might be that the BDP concentration in the drug–excipient solution with a low mass ratio was high, and many drug crystals emerged during the initial stage of the spraying process due to poorly soluble BDP. The fine particle size of the composite particles resulted from the enhanced nucleation rate. 

**Figure 5:**
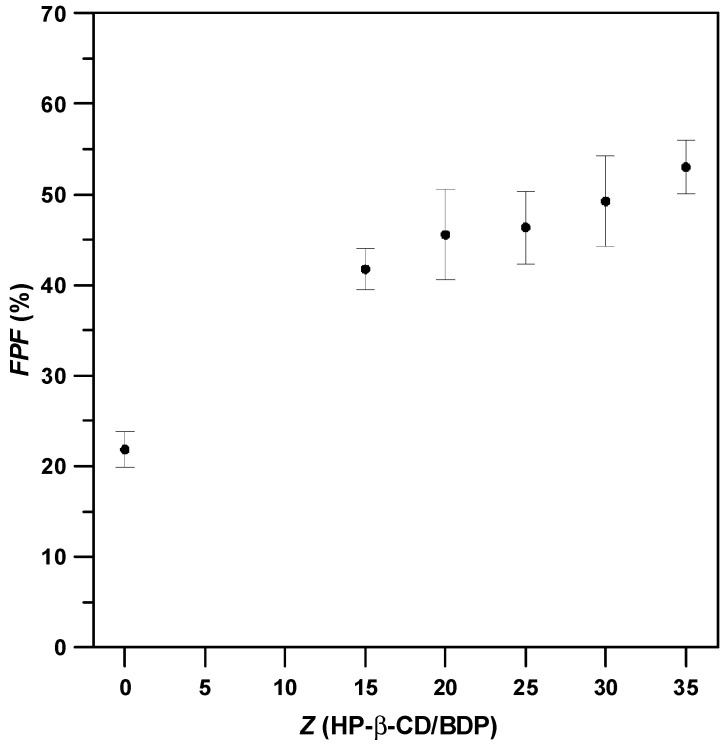
Variation of *FPFs* of drug–excipient composite powder with different HP-β-CD/BDP mass ratio (*Z*).

The aerodynamic performance of composite particles with different *Z* values is shown in Figure 5, indicating that the *FPF* values increased with the mass ratio (*Z*) of the composite particles. For samples with high mass ratio, BDP drugs were uniformly distributed in the composite particles and could be delivered to the lower respiratory system. Composite particles with a mass ratio of 35 (run #B7) exhibited excellent aerodynamic performance with an *FPF* value 2.4 times higher (run #B7, 52.2 ± 3.0%) than that of the as-received BDP (run #B1, 21.8 ± 2.0%), which could be attributed to their small *MMAD* and good flowability (low *H_R_*).

### 3.4. In Vitro Dissolution Performance of the Drug–HP-β-CD Formulation

As presented in Table 3 (runs #B3–#B7), the drug content in different drug mass ratios (*Z*) of the drug–HP-β-CD composite particles was close to the theoretical drug content (97–100%), which indicates that a solid dispersion of drug–HP-β-CD composite particles could be produced through SAA. The drug composite particles produced by SAA were uniformly dispersed as solid solutions and were conducive to precise drug formulation for DPIs.

**Figure 6:**
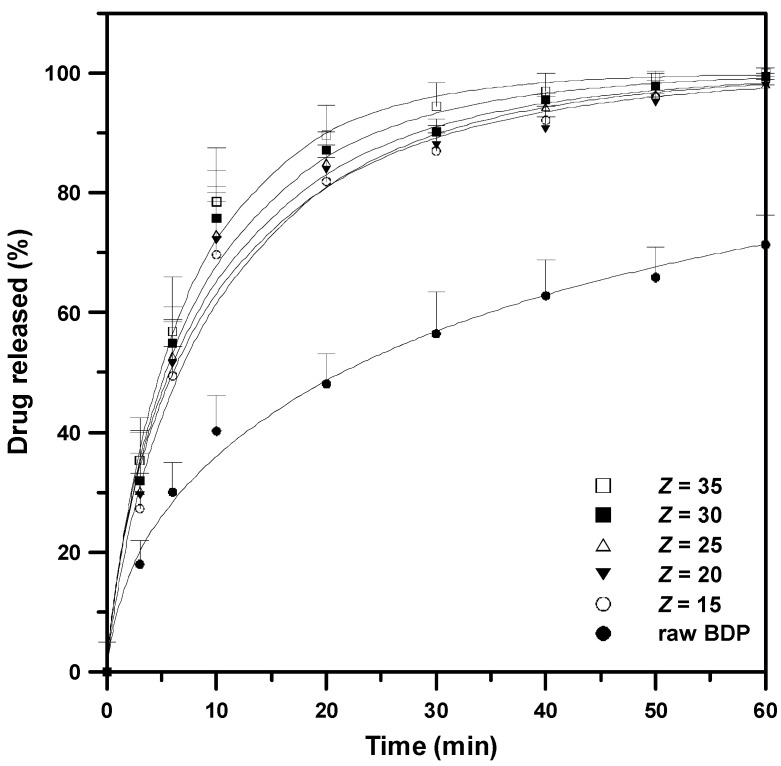
Dissolution profiles of drug–excipient composite powder with different HP-β-CD/drug mass ratio (*Z*): (●) as-received BDP, #B1; (◯) *Z* = 15, #B3; (▼) *Z* = 20, #B4; (△) *Z* = 25, #B5; (■) *Z* = 30, #B6; (□) *Z* = 35, #B7.

The in vitro dissolution test of the as-received BDP and drug–HP-β-CD composite particles with different mass ratio was performed in 0.05 M phosphate buffer saline (PBS, pH = 7.0) dissolution medium, as shown in Figure 6. The as-received BDP requires 36 h for complete drug release, indicating a poorly soluble drug. The dissolution rates of the drug–HP-β-CD composite particles with all drug mass ratios (*Z*) are higher than those of the as-received BDP. The drug composite particles produced using SAA release more than 80% of the drug in 20 min, and the drug in the composite particles is completely released in 60 min, indicating that the water-soluble HP-β-CD excipient can effectively improve the dissolution rate of poorly soluble drugs. The drug release profiles in this study were correlated using the Weibull model, and the regression parameters are presented in Figure 6 and Table 4. The in vitro dissolution test of the drug–HP-β-CD composite particles with all drug mass ratios (*Z*) were well correlated with the Weibull model (*r^2^* ≥ 0.97), and the estimated time for 63.2% drug release (*T_d_*) from the drug–HP-β-CD composites (*Z* = 35) can be reduced to 7.5 min, which represents an inhaled immediate-release formulation.

### 3.5. Solid Characterization

**Figure 7:**
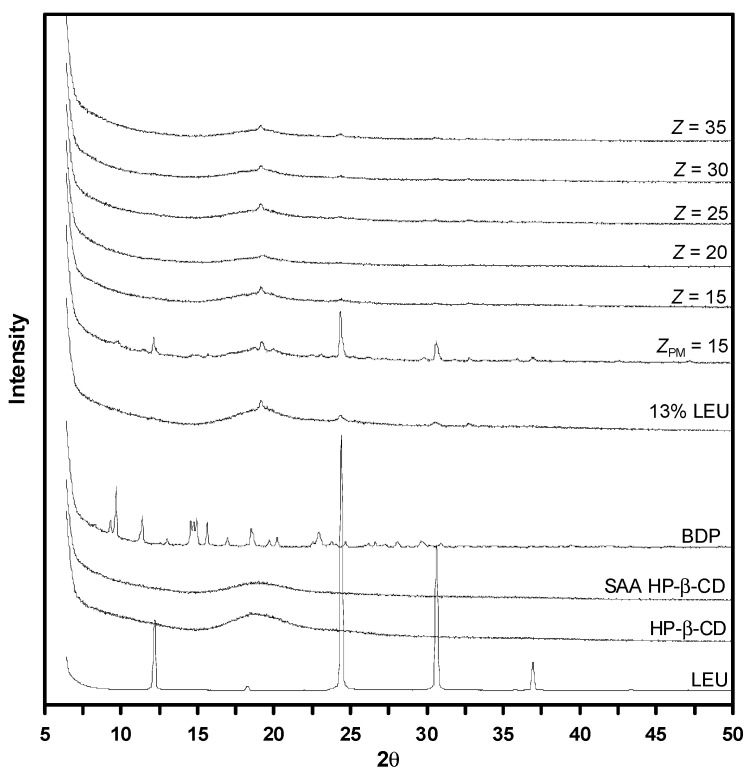
XRD patterns of HP-β-CD and drug–excipient composites produced through SAA.

Figure 7 shows the XRD patterns of the drug–HP-β-CD composite particles produced by SAA at different mass ratios (*Z*). A broad peak with low intensity can be observed for the as-received HP-β-CD and HP-β-CD particles produced by SAA (denoted as SAA HP-β-CD in Figure 7), indicating their amorphous morphology. The characteristic peaks of crystalline LEU appear at 12.1°, 18.4°, 24.4° and 30.6° [37]. The XRD pattern of the HP-β-CD particle sample with 13 mass% LEU exhibits a tendency of 20° characteristic peak. This phenomenon has been reported in many studies on the particles produced by the spray drying method and is attributed to the result of the crystallization of LEU on the surface of the particles [11,38,39]. The FESEM images also confirmed the presence of LEU crystals on the surface of the drug–HP-β-CD particles (Figure 4b). The as-received BDP shows characteristic peaks at 9.6°, 11.4°, 14.9° [40]. The sample prepared by physical mixing (*Z_PM_* = 15) also exhibits these three characteristic peaks of BDP and the characteristic peaks of LEU (12.1°, 20°, 24.4° and 30.6°). In the XRD patterns of the composite particles with different mass ratios (*Z*) produced by SAA, except for the obvious characteristic peaks of LEU, the characteristic peaks of BDP are significantly weaker than those observed in the sample prepared by physical mixing (*Z_PM_* = 15), indicating that the composite particles produced by SAA with different mass ratios (*Z*) tend to be amorphous.

**Figure 8:**
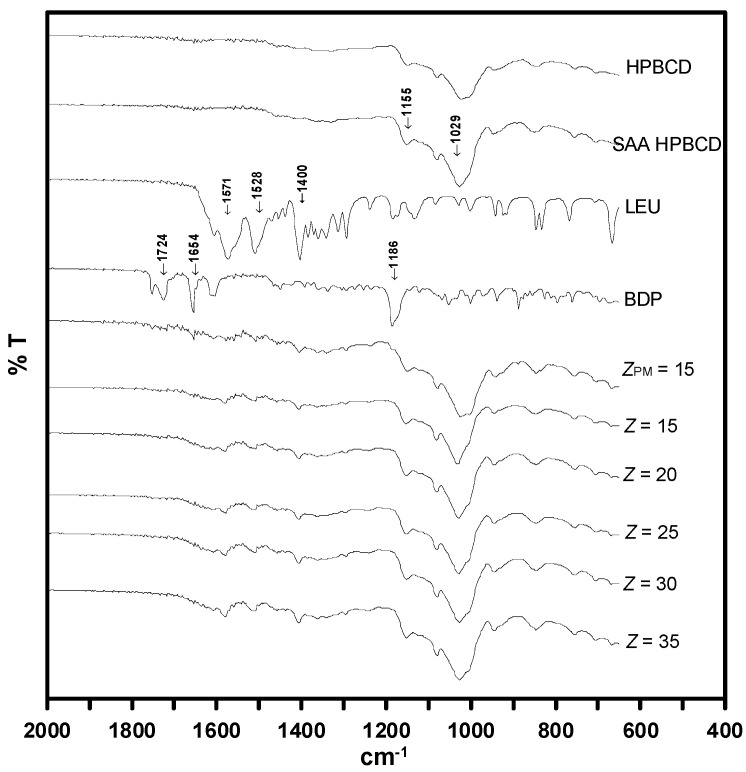
FTIR analysis results for HP-β-CD and drug–excipient composites produced through SAA.

The FTIR spectra (Figure 8) displayed characteristic peaks at 1155 cm^−1^ and 1029 cm^−1^ that were assigned to the C-O vibration and C-H group of H-β-CD, respectively, and the same patterns as those of the as-received HP-β-CD and H-β-CD particles produced by SAA. The spectrum of LEU showed intense absorption peaks at around 1571 cm^−1^ and 1400 cm^−1^, corresponding to the asymmetric and symmetric stretching modes of vibration of the COO– ion group, respectively, and the peak at 1528 cm^−1^ is indicative of the N-H^+^ stretch. The FTIR spectrum of BDP exhibited conjugated and non-conjugated C=O stretching bands at 1724 and 1654 cm^−1^, respectively. C=C stretching was observed at 1615 and 1608 cm^−1^, and the C-O bands were observed at 1186 cm^−1^ [41]. These bands were observed in the spectrum of the physical mixture sample (*Z_PM_* = 15). Thus, the characteristic peak of BDP almost disappeared in the drug-H-β-CD composite particles, indicating that BDP was included by H-β-CD or the formation of an inclusion complex.

**Figure 9:**
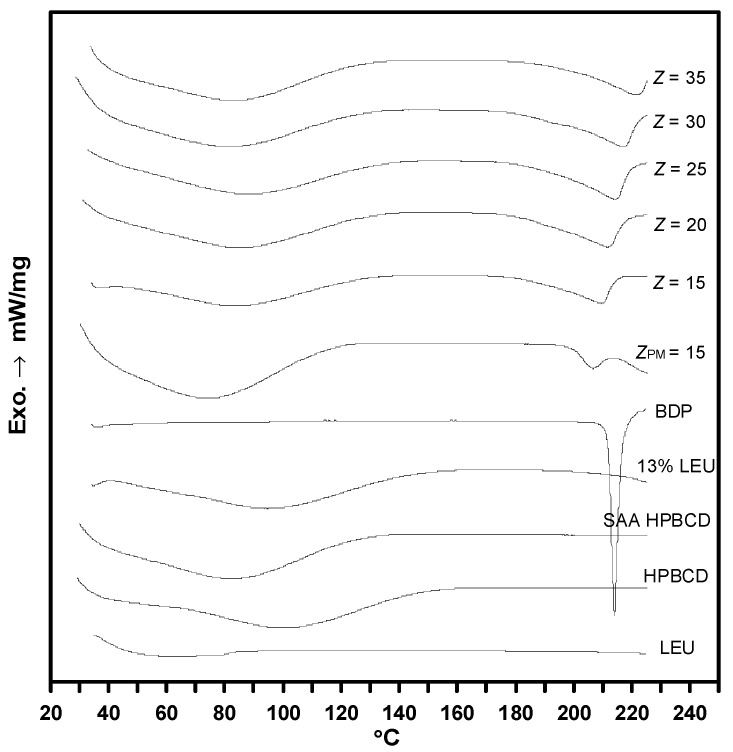
DSC analysis results for HP-β-CD and drug–excipient composites produced through SAA.

DSC was used to verify the inclusion complex formed between the drug and H-β-CD. When a guest molecule (drug) is embedded in the cyclodextrin cavity, the guest’s melting point, boiling point, or sublimation point usually shifts to a different temperature or disappears within the temperature range of cyclodextrin decomposition [3]. Figure 9 shows DSC results of the as-received HP-β-CD, SAA–HP-β-CD, and drug–excipients composite particles with different mass ratios (*Z*). The melting point of the as-received BDP drug is 213 °C, and the amorphous form of HP-β-CD exhibits a broad dehydration endothermic peak (50–125 °C) and subsequent thermal decomposition (240–400 °C) [42]. The physical mixing (PM) sample shows a broad dehydration endothermic peak of HP-β-CD and the melting point of BDP decreases to 205 °C. This implies that there was a partial interaction between BDP and HP-β-CD during the PM process that decreased the crystalline properties of the drug. Similar results have also been observed for β-CD/salbutamol composites produced by PM [43]. At the same mass ratio, the BDP–HP-β-CD composites (*Z* = 15) have no obvious BDP melting peak, compared with the physically mixed sample (*Z_PM_* = 15), indicating that the BDP–HP-β-CD composites produced by SAA form an amorphous inclusion complex.

Similar results were observed in the XRD and FTIR analyses (Figure 7 and Figure 8). Moreover, the dehydration endothermic peak area of the BDP–HP-β-CD composites is significantly low, indicating that the water in the HP-β-CD cavity is replaced by BDP. As the Z value increases, that is, the HP-β-CD content in the composites increases, drug–HP-β-CD composites tend to become amorphous inclusion complexes and the dissolution rate of the poorly soluble BDP drug can be enhanced, which is consistent with the results of the aforementioned dissolution experiment.

## 4. Conclusions

The phase solubility method was used to investigate the enhanced solubility properties of the poorly soluble BDP drug using HP-β-CD and ethanol. Aqueous ethanol increased the solubility of hydrophobic BDP because of the increased lipophilicity of the medium. The addition of HP-β-CD further enhanced the solubility of BDP, and the phase solubility profiles for BDP exhibited an A-type diagram. Inhalable drug–excipient (BDP–HP-β-CD) composite particles were successfully produced using a one-step SAA process. The conducive conditions for producing fine spherical particles with excellent aerosolization performance were 54.2% (*w/w*) aqueous ethanol as the solvent and addition of 13.0 mass% of the dispersion enhancer leucine. The effect of the mass ratio of HP-β-CD to BDP (*Z*) on the in vitro aerosolization and in vitro dissolution performance of BDP–HP-β-CD composite particles was evaluated. Drug–excipient composites with a mass ratio of 35 (run #B7) exhibited excellent aerodynamic performance, with an *FPF* value 2.4 times higher (run #B7, 52.2%) than that of as-received BDP (run #B1, 21.8%). The dissolution experiment indicated that water-soluble excipient (HP-β-CD) could effectively enhance the dissolution rate of BDP from composite particles. The time for complete drug release from the drug composite powder decreased to 60 min compared to 36 h for the as-received BDP. This study suggests that BDP–HP-β-CD composites produced through SAA can be used in immediate-release DPI formulations to improve the bioavailability of poorly water-soluble inhalation drug.

## Figures and Tables

**Table 1:** *CE* values calculated from the phase solubility profiles of BDP in HP-β-CD solution with different concentrations of co-solvent.

EtOH (%, *w*/*w*)	Complexation Efficiency (*CE*)	*R^2^*
0	0.00181	0.99
0.4	0.00197	0.99
4.0	0.00139	0.99
8.1	0.00149	0.99
16.3	0.00158	0.99
25.3	0.00154	0.96
44.2	0.00488	0.95
54.2	0.0143	0.98

**Table 2:** Parameters of Equation (3) for expressing BDP solubility as a function of both the concentrations of HP-β-CD and ethanol.

Parameter Symbol	Calculated Parameters
*σ* (mM^−1^)	0.3200
*K_b_* (mM^−1^)	3481.6
*K_t_* (mM^−2^)	220.61
*ρ_b_* (mM^−1^)	0.4863
*ρ_t_* (mM^−1^)	0.1704
*R^2^*	0.93

**Table 3:** In vitro aerosolization performance and drug content of drug–excipient composites with different mass ratio (*Z*) produced through SAA process.

Run	Z	C_BDP_	C_LEU_	ED	FPF	MMAD	Drug Content	*d_no_*	*d*_4,3_	*ρ_tap_*	*H_R_*
		mg/mL	mg/mL	%	%	µm	%	µm	µm	g/cm^3^	-
B1 ^1^	-		0	97.4	21.8 ± 2.0	3.95	-	-	-	-	-
B2	15	0.67	0.1	95.8	25.5 ± 1.0	3.43	6.20 ± 0.1	-	-	0.22 ± 0.01	1.44 ± 0.03
B3	15	0.67	1.5	97.4	41.8 ± 2.3	2.70	5.53 ± 0.1	1.20 ± 0.20	1.41 ± 0.20	0.20 ± 0.02	1.39 ± 0.01
B4	20	0.5	1.5	96.5	43.8 ± 5.0	3.13	4.13 ± 0.2	1.39 ± 0.15	1.46 ± 0.30	0.21 ± 0.02	1.38 ± 0.02
B5	25	0.4	1.5	99.5	45.6 ± 4.0	2.52	3.34 ± 0.1	1.45 ± 0.20	1.53 ± 0.25	0.20 ± 0.01	1.33 ± 0.02
B6	30	0.33	1.5	94.2	49.2 ± 5.0	2.22	2.79 ± 0.3	1.46 ± 0.22	1.55 ± 0.15	0.21 ± 0.02	1.36 ± 0.04
B7	35	0.29	1.5	98.4	52.2 ± 3.0	2.48	2.37 ± 0.2	1.49 ± 0.20	1.71 ± 0.18	0.19 ± 0.01	1.30 ± 0.05

^1^ B1: as-received BDP.

**Table 4:** Correlation results of the in vitro dissolution profiles of drug–HP-β-CD composite particles with different mass ratio (*Z*) produced using SAA.

Run	*Z*	Weibull ^1^			
		*a*	*b*	*r* ^2^	*T_d_* ^2^
B1 ^3^	-	8.385	0.574	0.98	40.5
B3	15	6.473	0.791	0.98	10.6
B4	20	5.316	0.726	0.97	10.0
B5	25	5.328	0.750	0.98	9.3
B6	30	5.407	0.788	0.98	8.5
B7	35	5.445	0.843	0.98	7.5

^1^ Weibull model: $m=1-\exp\left[\frac{-t^{b}}{a}\right]$. ^2^ Estimated time (min) of 63.2% drug released from the Weibull model, $T_{d}={\left(a\right)}^{\left(\frac{1}{b}\right)}$. ^3^ As-received BDP.

## Data Availability

Not applicable.
